# Natural Dibenzo-α-Pyrones: Friends or Foes?

**DOI:** 10.3390/ijms222313063

**Published:** 2021-12-02

**Authors:** Georg Aichinger

**Affiliations:** Laboratory of Toxicology, Department of Health Sciences and Technology, ETH Zurich, 8092 Zurich, Switzerland; georg.aichinger@hest.ethz.ch

**Keywords:** functional nutrition, emerging contaminants, urolithins, mycotoxins, chemoprevention, neuroprotection, DNA damage, estrogenicity, microbiome, bioactives

## Abstract

Natural dibenzo-α-pyrones (DAPs) can be viewed from two opposite angles. From one angle, the gastrointestinal metabolites urolithins are regarded as beneficial, while from the other, the emerging mycotoxin alternariol and related fungal metabolites are evaluated critically with regards to potential hazardous effects. Thus, the important question is: can the structural characteristics of DAP subgroups be held responsible for distinct bioactivity patterns? If not, certain toxicological and/or pharmacological aspects of natural DAPs might yet await elucidation. Thus, this review focuses on comparing published data on the two groups of natural DAPs regarding both adverse and beneficial effects on human health. Literature on genotoxic, estrogenic, endocrine-disruptive effects, as well as on the induction of the cellular anti-oxidative defense system, anti-inflammatory properties, the inhibition of kinases, the activation of mitophagy and the induction of autophagy, is gathered and critically reviewed. Indeed, comparing published data suggests similar bioactivity profiles of alternariol and urolithin A. Thus, the current stratification into hazardous *Alternaria* toxins and healthy urolithins seems debatable. An extrapolation of bioactivities to the other DAP sub-class could serve as a promising base for further research. Conclusively, urolithins should be further evaluated toward high-dose toxicity, while alternariol derivatives could be promising chemicals for the development of therapeutics.

## 1. Introduction

**Figure 1:**
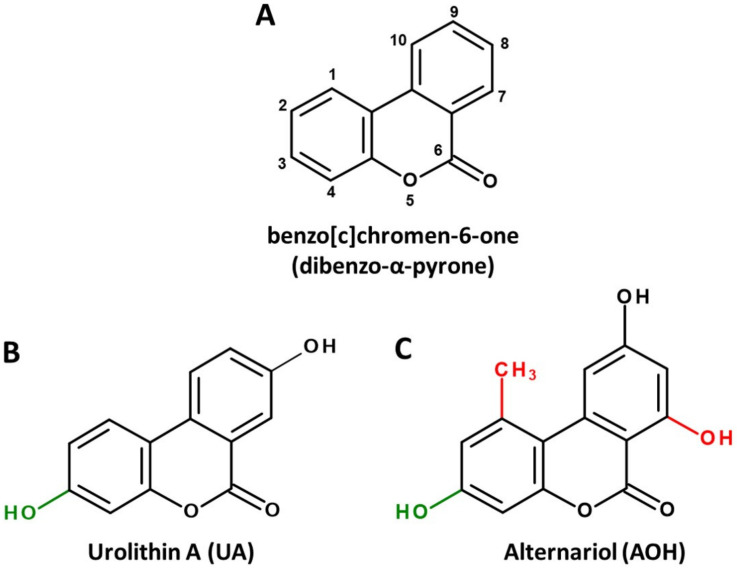
Chemical structures of the basic DAP scaffold (**A**), as well as two signature representatives of natural DAP derivatives: the bacterial polyphenol metabolite urolithin A (**B**) and the *Alternaria* mycotoxin alternariol (**C**). The hydroxy group at C1 (marked green) is a common feature of most natural DAPs. Methylation of C5, as well as hydroxylation of C11 (marked red), are common in biosynthesized DAPs but are not featured in ellagitannin biodegradation products.

Dibenzo-α-pyrone (DAP, Figure 1A) is the basic scaffold of a group of naturally occurring chemicals, which are mainly formed by microbial species, such as bacteria or filamentous fungi. When substituted with multiple hydroxy groups, those metabolites belong to the chemical class of polyphenols, from which many representatives are regarded as beneficial for human health, mostly due to anti-oxidative and chemopreventive effects [1]. This also applies to some polyphenolic DAP derivatives. For example, urolithin A (UA, Figure 1B), a metabolite formed by ellagitannin-degrading gut bacteria, is extensively researched and marketed as a health-promoting agent in the scope of functional foods [2] or even as a therapeutic agent to improve muscle health [3].

However, there seems to be another side to the story. DAPs biosynthesized by food-contaminating molds, such as mycotoxin alternariol (AOH, Figure 1C), are regarded as potential carcinogens due to their ability to damage the DNA and to potentially induce endocrine-disruptive effects [4,5]. Given the striking structural similarity of these natural DAPs, some rather uncomfortable questions present themselves. Is the division into healthy urolithins and toxic AOH derivatives justified due to distinct bioactivity, or is current research on natural DAPs incomplete due to the different viewpoints formed depending on the source of origin? Could a chemical hazard be hidden in beneficial antioxidants? Can positive health effects be attributed to certain mycotoxins? Do the different substitution patterns of hydroxy and methyl groups result in distinct biological activities, or do we merely observe beneficial effects at moderate doses of exposure and toxic effects at high doses (which seems to be a steady companion in research on polyphenols [6,7])?

This brief review aims at comparing the available literature on urolithins and mycogenic DAPs and focuses on the overlapping area of toxicology and functional nutrition, in order to provide a solid base for a more holistic research approach on DAP bioactivity.

## 2. Microbial Sources and Associated Structural Peculiarities

There are isolated reports of DAP derivatives being formed by plants [8], but the two main ways that they are produced in or from food commodities are (a) the complete biosynthesis as secondary metabolites of molds or (b) the biodegradation of ellagitannins by intestinal bacteria. The biosynthesis pathway is described mainly for filamentous fungi, particularly the genus *Alternaria*. A polyketide synthase encoded by the *pksJ* gene was found to be critical for the production of the two most prevalent DAPs, AOH and alternariol 9-methyl ether (AME) by *Alternaria alternata* [9]. Furthermore, the production of these and similar DAPs was also reported in other *Alternaria* [10], *Acremonium* [11], *Cephalosporum* [12] and *Hyalodendriella* spp. [13], all described as endophytic molds. Mycogenic DAPs are frequently reported in mold-contaminated grains, fruits, vegetables, etc., and are regarded as food contaminants [14]. As data suggest that they could be responsible for potentially toxic effects, but there are no regulations for maximum contamination levels yet around the globe, AOH and AME are considered to belong to the class of emerging mycotoxins [15].

On the other hand, DAPs that derive from the biodegradation of ellagitannins are uniformly referred to as urolithins. After ingestion, ellagitannins are hydrolyzed by bacteria carrying tannase enzymes to yield ellagic acid [16], which is further catalyzed by a currently unidentified lactonase/decarboxylase enzyme to the 3,4,8,9,10-pentahydroxy-DAP, urolithin M-5 (UM5). From the latter, all other urolithins are formed by subsequent dehydroxylation reactions that are catalyzed by currently unidentified enzymes [2]. However, a few bacterial species that are able to carry out at least a part of these reactions were already discovered. *Gordonibacter pamelae* and *Gordonibacter urolithinfaciens*, two species belonging to the strictly anaerobic family of *Eggerthellaceae*, were reported to decompose ellagic acid and perform dehydroxylations to sequentially yield UM5, urolithin M-6 and urolithin C (UC), the latter being the final metabolite [17]. Recently, another *Eggerthella* species, *Ellagibacter isourolithinfaciens*, was isolated from a human gut microbiome and observed to be capable of further dehydroxylating UC to isourolithin A [18,19]. Another study found *Bifidobacterium pseudocatenulatum* INIA P815 to produce UA and UB under certain growth conditions [20]. In complex microbiomes obtained from human feces, high interindividual differences were observed in the activity of the human microbiome, which allows for its stratification into three main groups [2]. Metabotype 0 (accounting for approximately 10–15% of the population) does not produce urolithins from ellagic acid. In urolithin producers, the final metabolites are either UA (metabotype A) or isourolithin A and urolithin B (UB) (metabotype B) [21].

Notably, the common precursor molecule UM5 predetermines that DAPs deriving from ellagic acid breakdown are only substituted with hydroxyl groups and are not functionalized at C1, C2 and C7 (Figure 1B). This is in stark contrast to biosynthesized DAPs, where substitutions at those positions, particularly the methylation of C1 and the hydroxylation of C7, are the norm (Figure 1C). In addition, based on current knowledge, UA and UB are not further metabolized by microbes, while for biosynthesized DAPs the methylation of functional hydroxy groups is common. For example, AOH is naturally produced as a mixture with AME, probably increasing its bioavailability and potentially its adverse effects [5].

## 3. Pharmacokinetics

Animal data on pharmacokinetics of urolithins and *Alternaria* toxins are only comparable to a limited extent due to differences in used species and experimental setups. However, according to a quick survey using the SwissADME quantitative structure–activity relationship (QSAR) tool [22], the bioavailability of major urolithins and fungal DAPs is predicted to be very similar (Table 1). UA and AOH, as well as UB and AME, share a comparable lipophilicity, and all four compounds have a 0.55 probability to be at least 10% bioavailable from oral uptake in rats, referred to as “bioavailability score” [23]. One exception is the blood brain barrier (BBB) permeation that is predicted only for UA/UB, not for AOH/AME (Table 1), which might be of high interest in the scope of neuroprotective effects that are proposed for UA. In line with this prediction, the presence of UA in mammalian brains was recently confirmed [24], while AOH was not reported to reach the brains of exposed mice in another study [25].

Corresponding with the predicted bioavailability score, a recent study on Sprague-Dawley rats found approximately 90% of orally consumed AOH and AME to be excreted via the feces [26]. Based on physico-chemical similarities, it seems reasonable to expect corresponding total uptake ratios for UA and UB.

Hepatic metabolism of natural DAPs is generally assessed qualitatively and lacks the application of state-of-the-art quantitative tools as of yet. Nevertheless, their biotransformation seems to be comparable, with rapid glucuronidation to less bioactive phase II metabolites as the main pathway of hepatic clearance [27,28].

Taken together, it seems very unlikely that differing pharmacokinetics cause a significantly distinct in vivo bioactivity of methylated and non-methylated DAPs.

## 4. Bioactivity Profiles

To address the question of whether significant differences exist between those compounds in the impact on human health, one must ask if the additional functionalization of AOH/AME, particularly the methylation of the DAP scaffold, might serve as a driving force of toxicity. Thus, the upcoming section will focus on comparing respective bioactivity data of AOH/AME and UA/UB as representative metabolites of each natural DAP class.

### 4.1. Topoisomerase Poisoning and Genotoxicity

In two-digit micromolar concentrations, AOH and AME are well described to act genotoxic in human cancer cell models by poisoning topoisomerase (topo) II, an enzyme critical to the maintenance of DNA integrity during replication and transcription [29,30]. To a lesser extent, the induction of oxidative stress might play a role in genotoxicity as well [31]. To act as topo poison, a molecule has to stabilize the so-called “cleavable complex” between the enzyme and DNA, preventing the ligation of a previously induced gap in the phosphate backbone of the DNA, which may then persist as a strand break [32].

Published data do not suggest that UA or UB might poison topoisomerases and exert corresponding in vivo genotoxicity. UA was found to increase micronuclei formation in cells that were exposed to concentrations of approximately 5 μM for 20 h, but did not cause genotoxicity in vivo or mutagenicity in the Ames test, which is why UA passed the general safety assessment for application as a food supplement [33]. Furthermore, ellagic acid and UM5 were reported to catalytically inhibit topo II by competing with ATP at sub-micromolar concentrations [34], a biological activity that might lead to an increased number of DNA strand breaks at high concentrations due to an impaired management of torsional stress [35]. UA and UB were found to be inactive toward topo II up to 5 μM in decatenation assays [34]. However, it should be noted that with the same method, AOH was found to inhibit topo II only at concentrations above 10 μM [30]. Thus, the testing of urolithins for interactions with topo II seems incomplete, and it should be encouraged to apply higher concentrations and methods to assess topo poisoning, e.g., the in vivo complex of enzyme assay [36].

With regards to genotoxicity, AOH and UA might exert similar biological effects, even if the published data are not fully comparable. Both were shown to induce the formation of micronuclei in cultured cells of different origin, starting from comparable concentrations [33,37], and both were reported not to cause systemic genotoxicity in rodents [25,33].

Natural DAPs in general have not yet been studied regarding potential genotoxic effects in the colon, which is the site where the highest doses of aglycons can occur—in up to millimolar concentrations in the case of UA [38]. For AOH, the gastrointestinal toxicity is considered one of the major knowledge gaps [5,25]. Thus, the respective testing of not only *Alternaria* mycotoxins, but also urolithins, should be encouraged to complement risk assessment.

### 4.2. Endocrine Activity

AOH and AME are reported as estrogen receptor (ER) agonists, resulting in related gene transcription and a growth stimulation of ER-positive cells [37]. Moreover, several metabolites of those compounds were predicted to act estrogenic in a mixed in silico/in vitro approach [39]. AOH was also found to interact synergistically with other xenoestrogens, such as the mycotoxin zearalenone or the soy isoflavone genistein [40,41], and to exert cumulative estrogenic effects with the plasticizer bisphenol A [42] towards estrogenicity. Furthermore, the two biosynthesized DAPs were also reported to activate other steroid receptors, such as the androgen (AR) [43] and progesterone receptor [44]. Together, these findings have sparked concerns about the endocrine-disruptive potential of *Alternaria* toxins [5].

For urolithins, similar concerns have been reported to some degree. UA in particular was found to have a high affinity for estrogen receptors, with IC_50_ values in ERα receptor binding assays being even lower as compared to the well-known dietary phytoestrogen genistein [45]. However, the stimulation of MCF-7 cell growth only took place at comparably high concentrations of about 40 μM, pointing toward a possible antagonistic mechanism. Furthermore, another study reported that 10 μM of UA induced ER-dependent gene transcription in human endometrial cancer cells, but, contradictorily, also suppressed cell proliferation, which might be linked to a differentiated activity toward ERα (antagonism) and ERβ (agonism) [46]. UA is also suspected to interact with AR activation, although the exact mechanism has not yet been elucidated. While a direct agonism or antagonism was not reported in luciferase reporter gene assays in MDA-kb2 cells [47], other studies found UA to decrease AR expression [48] and to increase the proportion of the receptor residing in the cytosol, leading to the hypothesis that this DAP might be a valuable bioactive toward the prevention of prostate cancer [49].

Furthermore, UA and UB were demonstrated to inhibit 17β-hydroxysteroid dehydrogenase (17β-HSD), an enzyme critical for the biosynthesis of the endogenous estrogen 17β-estradiol (E2) in a hybrid in silico/in vitro approach [50]. The resulting decrease in intracellular E2 levels might serve as an alternative explanation for the compounds’ anti-proliferative effects in breast cancer cells [50]. AOH and AME were not yet tested for effects toward an interaction with 17β-HSD. However, AOH lead to a reduced proliferation of human endometrial cancer cells, even as ER-mediated gene expression is activated [37]. While this effect can also be attributed to the onset of genotoxicity, the inhibition of 17β-HSD might be reconsidered as an alternative mechanism. Both fungal DAPs were reported to impair progesterone synthesis in porcine cells [51], potentially by interfering with 3-beta-hydroxysteroid dehydrogenase (3β-HSD).

Overall, it seems that natural DAPs generally exert a certain potential to act as steroid receptor agonists and a high potential to impair steroid biosynthesis, regardless of substitution patterns that are characteristic for their biological origin.

### 4.3. Inhibition of Casein Kinase 2

Casein kinase 2 (CK2) is a highly pleiotropic protein kinase whose overexpression is linked to pro-oncogenic processes [52] and anti-apoptotic effects in cancer treatment [53]. Thus, inhibition of CK2 has emerged as a therapeutic mode of action for overcoming drug resistance in cancers [54]. The DAP backbone seems to be a promising scaffold for this activity, as several representatives were predicted or reported to inhibit CK2. UA inhibited the enzyme with a IC_50_ of 0.39 μM and served as a precursor for the development of a much more potent inhibitor, its 4-bromo-derivative, that reached an IC_50_ of 0.015 nM [55]. For AOH, a similar IC_50_ (0.71 μM) regarding CK2 inhibition was observed in a cell-free assay, and the idea to base CK2-inhibiting drugs on its scaffold was ventilated [56]. In addition to the possible application in chemotherapy, a general chemopreventive effect of dietary CK2 inhibitors is currently discussed [57].

### 4.4. Mitophagy and Mitochondrial Health

Mitophagy is the cellular process of recycling damaged mitochondria that is central to mitochondrial health and of particular importance to highly stressed tissues, such as muscles [58]. It includes several pathways that can be influenced by extrinsic factors. It is well established that UA promotes mitophagy by stabilizing PTEN-induced kinase 1 (PINK1), responsible for recruiting and activating the protein Parkin, which in turn triggers the ubiquitination and thus degradation of mitochondrial proteins [59,60]. Exploiting this mechanism, the compound has even passed clinical trials as a promotor of mitochondrial and cellular health [61] and is marketed as a supplement to improve muscle health, particularly for elderly people [62].

AOH and AME have not yet been tested within the scope of inducing mitophagy, but given that the exact connection of chemical structure and PINK1 stabilization seems not very well elucidated, it might be a target for DAPs in general. Moreover, there is some evidence that an activation of the nuclear factor erythroid 2–related factor 2 (Nrf2) pathway, a process that not only UA [63] but also AOH and AME are capable of triggering [29], might play an additional role in the promotion of mitophagy [64].

### 4.5. Nrf2, Anti-Oxidative and Anti-Inflammatory Effects

In addition to the therapeutical application in the context of mitophagy, the propagation of urolithins as healthy dietary metabolites is based on their characterization as antioxidant and anti-inflammatory agents that have been extensively reviewed in recent literature [2,3]. The main mechanism behind their counteracting of oxidative stress is the activation of the Nrf2 pathway. The protein is bound to “Kelch-like ECH-associated protein 1” (Keap1) in the cytosol, which undergoes conformational changes to release Nrf2 in the presence of reactive oxygen species (ROS) or other electrophilic agents [65,66]. It then relocates into the nucleus, where it serves as a transcription factor inducing the expression of endogenous antioxidant agents and enzymes. Additionally, anti-inflammatory effects are mediated by Nrf2 via a crosstalk with NF-κB signaling [67]. Consequently, the beneficial compounds that trigger this pathway usually act slightly pro-oxidatively themselves and/or might even cause cellular oxidative stress at significantly higher concentrations.

Unfortunately, UA and UB have not yet been thoroughly tested for a pro-oxidative potential, but extensive data exist on their protective role against stressors, most commonly H_2_O_2_, which occurs in vitro at low micromolar concentrations [64,68,69]. On the contrary, the mycotoxins AOH and AME have not been tested for potential protective effects, but only for their direct pro-oxidative potential. In human cancer cells, these mycogenic DAPs lead to an increase in intracellular ROS levels at concentrations ≥5 µM, but they also cause an activation of Nrf2-related gene transcription at nanomolar doses [29]. Assessing a potentially corresponding ability to counteract oxidative stressors might be promising and should be encouraged, particularly in light of the Nrf2 activators being extensively tested as therapeutic agents for the treatment of type 2 diabetes mellitus [70].

Regarding a possible anti-inflammatory activity, UA/UB [71,72,73], as well as AOH/AME [74,75,76,77], have been reported to counteract pro-inflammatory stimuli in different cell models. Mechanisms that might play a role are the cholesterol-like intercalation into the cell membranes of macrophages that interferes with immunomodulatory receptors [78] or an interplay with NF-κB signaling to produce and release pro-inflammatory cytokines, potentially mediated again via Nrf2 activation [79].

### 4.6. Autophagy and Senescence

Autophagy is the process of degradation and recycling of cytosolic proteins of damaged cells, which is mostly associated with beneficial health effects, such as the prevention of cellular stress and tumor progression [79]. Several studies have reported the induction of this process after the exposure of different cells to DAPs of distinct sources. UA was found to induce this autophagy and to thereby inhibit metastasis-related biomarkers in colorectal cancer cells [80] and to protect neural cells from injury by decreasing endoplasmic reticulum (ER) stress [81]. Furthermore, there is some evidence suggesting that a part of the anti-inflammatory properties of UA might be related to increased autophagy in macrophages [82].

Interestingly, AOH was also reported to induce autophagy in macrophages, presumably via the mediation of ER stress that triggers the mTOR pathway [83]. However, in that study, a prolonged exposure of cells to AOH resulted in cellular senescence, a less desirable condition. From the two studies conducted on macrophages, it seems likely that UA and AOH exert similar effects toward the induction of autophagy in human cells.

### 4.7. Interactions with the Gut Microbiome

Large parts of ingested DAPs, such as AOH and AME, are excreted via the feces [26], and thus the gastrointestinal tract is probably the primarily exposed organ, which has sparked interest on potential interactions with the gut microbiome as an additional mode of action. A recent study addressed these questions and reported inhibitory effects of a complex mixture of *Alternaria* toxins on a multitude of bacterial strains and their ability to form biofilms [84]. However, as the extract that microbes were exposed to contained large amounts of mycotoxins with other chemical structures, a causal relationship with exposure to AOH/AME cannot be established from the published research. Likewise, experiments simulating the gastrointestinal tract revealed pomegranate extract to modulate the composition of the microbiome by increasing the prevalence of *Akkermansia* and *Gordonibacter*, particularly in the distal colon [85]. Furthermore, these changes seemed to have a direct enhancing impact on the formation of urolithins. Again, it seems difficult to draw a direct conclusion on the effects of DAPs on microbial communities due to the chemical complexity of the applied extract.

## 5. Conclusions

Taken together, the differentiation between “healthy” urolithins and “toxic” AOH derivatives seems to be a direct consequence of the reputation of their respective origins (superfoods vs. molds) and thus should be viewed critically. The two signature compounds of the respective groups, UA and AOH, are predicted to exert similar pharmacokinetic characteristics and share many biological activities, such as in vitro genotoxicity at high doses, the interaction with steroid receptor activation and steroid biosynthesis, the activation of the Nrf2 pathway, related anti-inflammatory effects and the induction of autophagy. However, blind spots on both sides prevent a full comparability of existing data.

On the one hand, the risk assessment of urolithins might not be fully completed yet, particularly regarding potential endocrine effects of higher doses that could hypothetically be reached via the application of pure urolithins as supplements. On the other hand—and somewhat counterintuitively—DAPs produced by *Alternaria* spp. and similar fungi might have a hidden potential in chemoprevention or as scaffolds for the design of therapeutic bioactives. A particularly relevant open question is whether AOH and AME would be able to promote mitophagy in a comparable way as their siblings of bacterial origin. Additionally, sub-genotoxic concentrations of mycogenic DAPs could hypothetically play a role in reducing cellular oxidative stress by activating the Nrf2 pathway, which could be utilized for the design of novel Nrf2-activating agents, e.g., for the treatment of diabetes mellitus.

It should be said that the discrepancies around research on DAPs might serve as an example of how research focus and chosen methodology can shape scientific understanding. A direct comparison of results for the two DAP classes is very challenging due to the different scientific fields (pharmacology vs. toxicology) and the respectively chosen experimentation. Nevertheless, such a comparative approach could allow for a prediction of possible biological targets for compounds of the other DAP class and thus could prove highly valuable in fueling corresponding research. In this light, it seems obvious that studies that test DAPs of different origin with a harmonized methodology would be of great value in evaluating the remaining toxicological or pharmacological questions.

## Figures and Tables

**Table 1:** Pharmacokinetic parameters of major natural DAPs. Shown are: octanol-water partition coefficients (PO/W), gastrointestinal (GI) adsorption, blood brain barrier (BBB) permeability and bioavailability score, as predicted by the SwissADME QSAR [21].

	log P_O/W_	GI Absorption	BBB Permeant	Bioavailability Score
UA	2.06	high	yes	0.55
UB	2.48	high	yes	0.55
AOH	2.17	high	no	0.55
AME	2.55	high	no	0.55

## Data Availability

Not applicable.
